# Development and preliminary validation of a fertility concept scale for Chinese post-00s college students based on thematic analysis

**DOI:** 10.3389/fpsyg.2026.1841927

**Published:** 2026-09-16

**Authors:** Lei Xiao, Xinrui Xiao, Zeyu Chen, Caihua Zhu, Yingmin Wu

**Affiliations:** School of Management, Chengdu University of Traditional Chinese Medicine, Chengdu, China

**Keywords:** fertility concept, post-00s college students, scale development, social network theory, thematic analysis, theory of planned behavior

## Abstract

**Background:**

China faces an increasingly acute risk of entering a “low-fertility trap,” and existing policy incentives have not produced the expected effects, underscoring the need to examine the micro-level psychological processes underlying fertility decision-making. Analyzing the fertility concepts of the post-00s generation is critical for predicting fertility behavior and informing targeted policies; however, suitable measurement tools remain lacking.

**Methods:**

Based on the Theory of Planned Behavior and Social Network Theory, a mixed-methods approach combining collection of publicly available online data, thematic analysis, cognitive interviews, and quantitative research was employed to develop a fertility concept scale tailored to Chinese post-00s college students. Data were collected through a pilot survey and a formal survey. Scale properties were evaluated using exploratory and confirmatory factor analyses, internal consistency testing, test–retest reliability, random split-half cross-validation, bootstrap analysis, and measurement invariance testing.

**Results:**

The final scale comprised 29 items across seven dimensions. Confirmatory factor analysis indicated good model fit (RMSEA = 0.059, CFI = 0.934, TLI = 0.925, SRMR = 0.051), with satisfactory internal consistency for each dimension (Cronbach's α > 0.8) and good test–retest reliability (ICC > 0.75). Random split-half testing and bootstrap analysis supported cross-sample stability, and the scale demonstrated measurement invariance across gender (ΔCFI ≤ 0.01, ΔRMSEA < 0.015).

**Conclusion:**

This scale has preliminarily demonstrated good reliability and validity, complementing existing instruments in terms of dimensional completeness, population applicability, and cultural adaptability. Its development process also offers methodological guidance for scale construction targeting internet-active populations and sociopsychological constructs characterized by strong intergenerational variation. After further testing of the scale's external validity and cross-sample stability, this instrument can be utilized for dynamically monitoring the fertility concepts among post-00s college students and the exploration of the mechanisms underlying the formation of their fertility intentions.

## 1 Introduction

China's total fertility rate was 1.00 in 2023, only slightly higher than that of South Korea (0.72), ranking second lowest among major global economies (Yan et al., 2025). China has entered a demographic phase characterized by low birth rates, low mortality rates, and low population growth (Wei et al., 2019), accompanied by accelerated population aging. Projections indicate that China's aging rate will rise from 10% to 30% within 40 years, whereas Western industrialized nations such as the UK, France, and the US took 100 years or longer to reach comparable levels (Yuan, 2022). Rapid aging leads to a shrinking labor force, weakens social innovation vitality and economic growth potential, increases payment pressures and operational burdens on social security systems, and poses severe challenges to socioeconomic development (Lei and Chen, 2025).

To address persistently low birth rates, China has gradually relaxed birth restrictions since 2011 and further established a policy support system after 2021. Following the implementation of these measures, the fertility potential among individuals born in the 1970s and 1980s was realized (Qing et al., 2021). The number of births in China exhibited a steady upward trend from 2011 to 2016, reaching a peak of 17.86 million in 2016. Since then, the number of births has declined; by 2025, it had fallen to 7.92 million—the lowest level since 1949—marking the first time it had dropped below 8 million, indicating that policy adjustments have not achieved the desired effect and that the overall trend of low fertility has not been reversed (Yang H. et al., 2023; Wang et al., 2024; Xu and Xu, 2024). This signifies that the fertility behavior of the primary childbearing cohort has undergone significant changes, making it imperative to focus on the fertility concepts and characteristics of the future main childbearing cohort in order to identify a breakthrough.

The post-00s generation will become the main reproductive cohort over the next 5–10 years. Currently in a critical period for forming their reproductive outlook, their fertility concepts will have a profound impact on the future trajectory of China's population structure. Among them, college students—owing to their higher education levels, openness to new ideas, and progressive values—are emerging as pioneers in societal attitudinal change (Yang and Li, 2021). Evaluating the fertility concepts of post-00s college students can help accurately identify public needs and facilitate proactive responses, thereby providing a forward-looking perspective for predicting fertility trends and formulating targeted fertility policies. However, few standardized measurement tools have been developed for this population, and existing scales are inadequate in terms of dimensional completeness, population suitability, and cultural adaptability, which hinders in-depth research on fertility concepts.

## 2 Theoretical foundation and research progress

### 2.1 Theoretical foundation

This study integrates the Theory of Planned Behavior (TPB) and Social Network Theory (SNT) to conceptualize the fertility concepts of post-00s college students. TPB posits that attitudes, subjective norms, and perceived behavioral control shape fertility intentions (Ajzen, 1991), whereas SNT is embedded within TPB to delineate the multiple sources of subjective norms (Figure 1).

**Figure 1:**
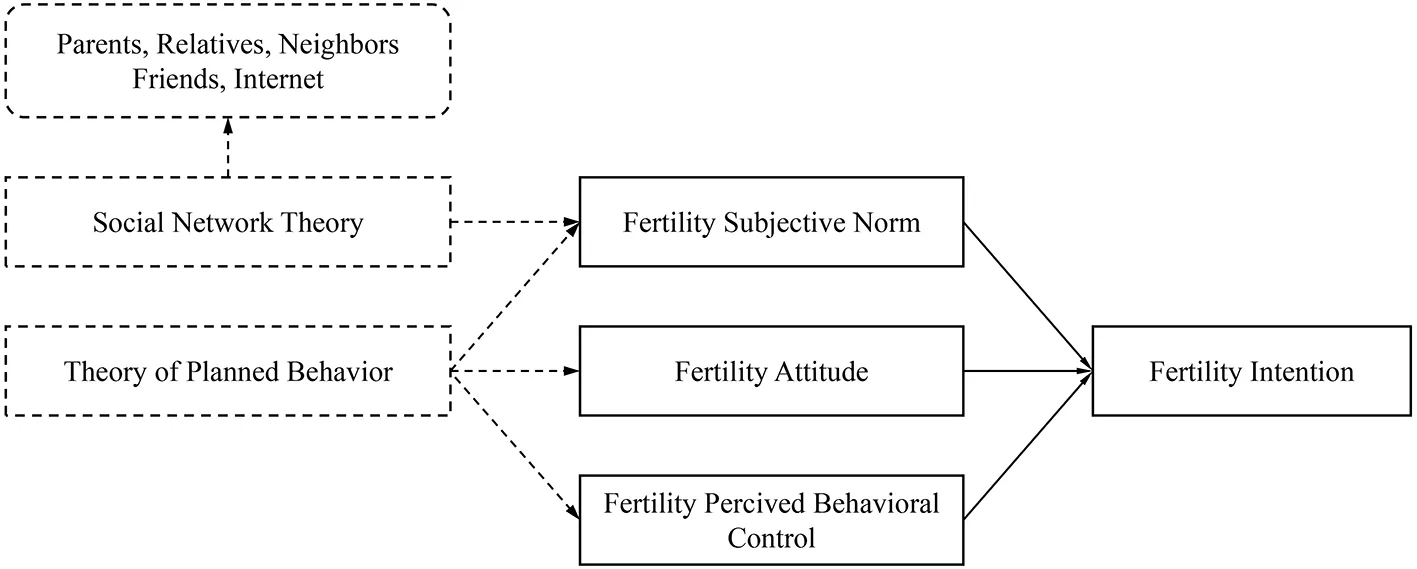
Theoretical framework for the fertility concept of college students.

Within TPB, subjective norms are typically treated as a unidimensional construct. However, in complex social environments, “significant others” are not homogeneous. Subjective norms can instead be differentiated into traditional and modern sources (Chukwudeh and Oduaran, 2024; Sahraei et al., 2024). These sources may convey divergent or conflicting expectations, which individuals can recognize and navigate. Social network theory provides the theoretical basis for this differentiation. Spouses, peers, older relatives, and younger family members exert sustained influence on fertility behavior (Yu and Liang, 2022; Chukwudeh and Oduaran, 2024), whereas internet-derived information shapes the value systems and behavioral patterns of post-00s youth (Wang, 2021). Accordingly, within the TPB framework, subjective norms can be decomposed into subdimensions linked to distinct social networks, including parents, relatives, neighbors, friends, and online sources (Yu and Liang, 2022; Huang, 2023; Chukwudeh and Oduaran, 2024; Zhang and Zhao, 2024; Xiao and Jiang, 2026). This specification captures discrepancies across normative sources and more accurately represents the heterogeneous normative environment experienced by post-00s youth.

### 2.2 Post-00s college students exhibit distinct characteristics in their views on fertility

Existing fertility-related research has primarily focused on married couples with children, emphasizing analyses of actual reproductive decision-making and policy responses (Ning et al., 2022; Yang Y. et al., 2023). Studies specifically targeting unmarried, childless college students remain relatively limited. Compared with earlier generations, post-00s college students demonstrate distinct characteristics in their fertility views. They navigate a complex era with diverse information channels (Hu et al., 2022) and encounter new ideological trends such as “DINKs” (Gao, 2021). Unlike previous generations, who regarded childbearing as a natural life process, they prioritize balancing reproduction with personal development (Mao et al., 2024). However, since post-00s college students have not yet entered the stages of marriage and childbearing, they do not face the practical constraints of reproductive decisions or the real pressures of parenting. Their understanding of fertility largely derives from others or from online sources (Ke et al., 2023; Lan et al., 2023). Consequently, their fertility concepts remain in a formative and developmental stage, exhibiting significant malleability. This particularity and complexity make the study of post-00s college students' fertility concepts both challenging and necessary, while current research efforts remain insufficient.

### 2.3 Fertility concept as one of the key research areas

Classical fertility theories explain mechanisms underlying low fertility through two major paradigms, structuralism and culturalism, corresponding respectively to family economics and social psychology (Wu, 2020; Song, 2023). The social psychology paradigm posits that shifts in social attitudes and values constitute the intrinsic roots of low fertility. Representative theories include the Second Demographic Transition (SDT) theory (Lesthaeghe, 2014), the deinstitutionalization theory (Cherlin, 2004), and the gender revolution theory (Goldscheider et al., 2015). SDT emphasizes that the rise of individualistic values promotes the deinstitutionalization of fertility, while deinstitutionalization theory focuses on individual roles in restructuring the institution of marriage. The gender revolution theory highlights how the diffusion of gender equality concepts cognitively reshapes fertility decisions.

The fertility concept is a locally distinctive Chinese construct that reflects individuals' fundamental views and attitudes toward childbearing within a specific historical and social context. As the core component of fertility culture, it serves both as an internal driver of fertility behavior and as a key force underlying changes in such behavior (Xu, 2008; Luo, 2008; Chen and Wu, 2008). Most scholars conceptualize fertility concept as a complex, multidimensional, and holistic construct that encompasses fertility intention, fertility attitude, and fertility motivation (Zou and Lai, 2024; Song, 2023; Sheng and Li, 2023; Luo, 2008).

The TPB provides a well-established framework for operationalizing and measuring such multidimensional psychological constructs. According to the TPB, fertility intention is determined by three core components: behavioral attitude, subjective norm, and perceived behavioral control (Ajzen, 1991). Fertility intention refers to an individual's desire to have children after considering various constraints and personal preferences. It represents subjective expectations regarding reproductive behavior and outcomes (Gu, 2011; Feng, 2017) and serves as the behavioral expression of fertility concepts (Yang and Wu, 2021; Zhao and Zhang, 2025; Feng and Zhang, 2002).

Fertility attitude reflects individuals' evaluative judgments regarding childbearing and provides the psychological foundation for the formation of fertility intention (Zu and Yang, 2026). Fertility motivation, in turn, represents individuals' deeper assessments of why they choose to have children and the value they assign to childbearing, encompassing both fertility purposes and fertility values (Sheng and Li, 2023). In addition, subjective norms and perceived behavioral control capture individuals' perceptions of social expectations regarding childbearing and their assessments of the feasibility of having children, respectively. Both factors are important determinants of fertility intention (Billari et al., 2009; Mencarini et al., 2015) and constitute core dimensions of fertility concepts.

Overall, fertility motivation, fertility attitude, and fertility intention form a progressive relationship extending from deep-seated values to behavioral tendencies. Subjective norms and perceived behavioral control further enrich the conceptual framework by incorporating external social influences and perceived individual capabilities. Therefore, developing a fertility concept scale grounded in the TPB is methodologically sound and operationally feasible.

### 2.4 Fertility-related measurement tools are poorly suited for the college students

Research on fertility issues primarily examines individuals' perceptions and tendencies regarding reproductive behavior through more specific constructs such as fertility motivation and fertility intention. Existing fertility intention scales fall into two major categories. The first type is designed for populations currently facing fertility decisions, such as married individuals, parents, or working adults. Billari et al. developed a fertility intention scale based on the TPB, measuring fertility attitudes through both costs and benefits. It evaluates subjective norms by considering the reproductive status and relational closeness of significant others, while perceived behavioral control is assessed through self-perceived readiness for childbearing and perceived control over life (Billari et al., 2009). Additionally, Mencarini et al. (2015), Li et al. (2019), and Iacovou and Tavares (2011) also developed fertility intention scales structured around the three core constructs of the TPB model. Another approach targets youth who are in the formative stage of their fertility concepts, often combining existing scales directly (Qian and Huang, 2024). However, this method suffers from inadequate cultural adaptation and fails to capture the specific characteristics of unmarried, childless populations. Qiao et al. (2024) developed a fertility intention scale specifically for Chinese female college students based on the TPB and TDIB models. This scale measures fertility intention, perceived barriers and supports related to childbearing, and feminist identity. Although it demonstrates high reliability and validity, it also exhibits dimensional overlap and incomplete coverage. Moreover, several questions are disconnected from the actual experiences of college students, which may prompt arbitrary responses and affect the accuracy of the measurement.

### 2.5 Solidification of scale development methodology

Mixed-method research that combines qualitative and quantitative approaches represents the mainstream paradigm for scale development. Core quantitative research techniques have achieved broad academic consensus. In contrast, qualitative methods offer greater flexibility in methodological choice, though the mainstream approach typically involves literature review, interviews, or expert consultation (Naghibi et al., 2019; Ashra et al., 2025). Among these, expert consultation leverages domain experts' knowledge but requires multiple iterative rounds, is time-consuming, and may deviate from the actual experiences of research participants, making it difficult to align with participants' cognitive logic and potentially leading to misjudgments regarding item appropriateness (Zhang et al., 2024).

In summary, although previous research has yielded substantial findings and made important contributions in the field of fertility concepts, several limitations remain:

(1) The generational distinctiveness of fertility concepts among post-00s college students has not been systematically elucidated;
(2) Existing measurement tools lack sufficient cultural adaptability, contain missing and overlapping dimensions, and fail to thoroughly explore the multidimensional composition of fertility concepts among Chinese post-00s college students;
(3) Scale development methodologies have become formulaic, failing to address the complexity of emerging research topics or the evolving challenges of real-world contexts.

Refining fertility concept measurement tools is therefore essential for clearly understanding the evolutionary trajectory, generational characteristics, and underlying structure of Chinese post-00s college students' fertility concepts. Such refinement holds significant academic and practical value.

### 2.6 Research aims

Based on an integrated theoretical framework combining TPB and SNT, this study developed a fertility concept scale tailored to Chinese university students born in the post-00s generation. The scale delineates the multidimensional structure of their fertility concepts, addressing limitations of existing measurement tools in dimensional completeness, population suitability, and cultural adaptability. It provides an integrated measurement framework and a standardized foundation for investigating the mechanisms underlying fertility intention development. In addition, it offers a standardized instrument for monitoring fertility concepts among Chinese post-00s college students.

## 3 Materials and methods

### 3.1 Collection of publicly available online data

This study identified 19 search keywords through a literature review, including marriage, delayed marriage and childbearing, pregnancy, fertility, child-rearing, having children, raising children, parenting, gender division of labor, cohabitation before marriage, premarital sex, the value of children, birth-pushing, and the motherhood penalty. Four representative Chinese social media platforms—TikTok, Rednote, Zhihu, and Bilibili—were selected as data sources. These platforms represent major online activity spaces for young people in China. Their complementary content formats (short videos, text–image posts, and in-depth discussions) and diverse user demographics (across genders and cognitive levels) enable the comprehensive capture of multidimensional information relevant to fertility concept.

Using web browsing and data collection methods, posts were collected from the four platforms between May 31, 2021, and September 2024, yielding 1,910 posts: 502 from TikTok, 623 from Rednote, 354 from Zhihu, and 431 from Bilibili. The collected data were imported into Excel for cleaning, including the removal of duplicate records, filtering of invalid elements (e.g., emojis and blank spaces), and exclusion of irrelevant or ambiguous comments, commercial promotional content, and data clearly originating from married individuals or parents. This process resulted in 85,691 characters of valid online text for subsequent thematic analysis.

The data collection plan for this study was approved, and the entire data collection process adhered strictly to academic ethical standards. Only user comments that were publicly available on the platforms were collected, platform access permissions were not exceeded, and the data collection process did not violate the robots.txt protocols or terms of use of the relevant websites. All web-based text data were fully anonymized, and personally identifiable information, including usernames, IDs, profile images, and geographic locations, was removed. Text involving personal privacy or intimate experiences was excluded from thematic analysis and indexing. The anonymized raw data cannot be used to identify any real-world users, thereby effectively minimizing the risk of personal information disclosure.

### 3.2 Thematic analysis

Thematic analysis was conducted according to the following procedures:

(1) The core constructs of the TPB were used to define the scope and direction of coding, thereby preventing excessive divergence during the coding of unstructured text.
(2) Two coders independently analyzed the cleaned online data using NVivo 11 Plus and extracted initial codes from the source material. For example, from the statement “I am hedonistic; I don't want to spend money on children,” content reflecting a comparison between self-indulgence and child-rearing was extracted and coded as “Focus on self-indulgence.”
(3) Themes were identified based on the initial codes. For example, statements coded as “Focus on self-indulgence” contributed to the theme “value prioritization,” whereas statements such as “Carry on the family line” contributed to the theme “value of children.”
(4) Coding consistency was evaluated using the Kappa coefficient, with values greater than 0.8 considered indicative of high agreement (Landis and Koch, 1977; Wen et al., 2018). This procedure helped reduce subjective bias. In addition, one-third of the reserved raw data were used for theoretical saturation testing to determine whether any new themes or information emerged.

When coding discrepancies, such as inconsistencies in code extraction or theme identification, the two coders discussed their respective rationales to reach a consensus. For cases in which consensus could not be achieved, the research team convened a focus group discussion and determined the final coding outcome with reference to the TPB framework and relevant literature.

This study used the TPB as the guiding analytical framework while incorporating expressions derived from the raw data to enrich the conceptualization of the core constructs. The identified themes were aligned with the theoretical framework while remaining grounded in the meanings embedded within the empirical data, thereby providing a foundation for integrating theory and data during subsequent scale development. The example comments presented in this study were paraphrased, and only abstract themes that cannot be traced to specific individuals are reported.

The identified themes were subsequently used as scale dimensions, and scale items were developed based on the initial codes (Table 1). For example, the item “Personal enjoyment and freedom are more important than raising children” was developed from the initial code “Focus on self-indulgence” and assigned to the “Value prioritization” dimension. Similarly, the item “Children can extend their parents' lives” was developed from the initial code “Carry on the family line” and assigned to the “Value of children” dimension.

**Table 1:** Example of thematic analysis.

Processed web text	Initial code	Themes	Items
I'm the type who likes to have fun, and I don't want to spend my money on kids.	Focus on self-indulgence	Value prioritization	Personal enjoyment and freedom are more important than raising children
I don't want to be a woman who spends all her time revolving around her children; I want to fulfill my own potential.	Pursuit of self-actualization		Achieving self-worth is more important than raising children
Raising children is about passing on life and being there for them, without expecting anything in return.	Carry on the family line Emotional reliance	Value of children	Children can extend their parents' lives Children can serve as emotional support for parents

Furthermore, drawing on social network theory and the literature, the study identified sources of subjective norms and developed measurement items for fertility intention dimensions through literature review and focus group discussions, balancing theoretical consistency with real-world contexts.

### 3.3 Interview

Generating scale items from publicly available social media data reduces social desirability bias, as users are unaware of their participation in research and their expressions more closely reflect their true views. However, because the demographic characteristics of commenters cannot be reliably verified and online expressions may be performative, cognitive interviews were conducted to assess whether the generated items aligned with post-00s college students' genuine understanding and expression of fertility concepts.

Using convenience sampling, 30 undergraduate and vocational college students from universities in Sichuan participated in cognitive interviews conducted in two phases, and all respondents subsequently participated in the pilot survey. Prior to the pilot survey, preliminary items were evaluated to identify and correct semantic ambiguities through think-aloud protocols and structured probing, ensuring clear, unambiguous, and non-leading wording. Items reflecting views of non-target groups or inconsistent with the cognitive frameworks of post-00s college students were identified and removed. Following the pilot survey, items that did not meet the criteria in reliability analysis and exploratory factor analysis were re-evaluated by examining the response process to determine the source of non-compliance. Items were revised and retained if issues arose from wording, or removed if they were not aligned with participants' concerns.

### 3.4 Questionnaire survey

#### 3.4.1 Questionnaire content

The Chinese Post-00s College Students' Fertility Concept Survey Questionnaire was designed to comprise two sections: a general information form and a fertility concept scale. The scale encompassed seven dimensions: value prioritization, value of children, family and neighborhood norms, peer and internet norms, self-perceived ability, pressure and cost, and fertility intention. A five-point Likert scale was adopted, where “1” → “5” represented “Strongly Disagree” → “Strongly Agree” and “Very Weak” → “Very Strong,” respectively. The dimensions of value prioritization, peer and internet norms, and pressure and cost were reverse-score.

#### 3.4.2 Sampling

This study selected Sichuan Province as the sampling frame and employed a combination of probability and non-probability sampling methods to obtain the sample. Using stratified sampling and PPS sampling based on university tier, student enrolment size, and geographic location, 16 sample institutions were randomly selected. These included 2 Double First-Class universities, 7 undergraduate institutions, and 7 junior colleges, covering five major regions: the Chengdu Plain, Northeast Sichuan, South Sichuan, Northwest Sichuan, and the Panzhihua–Xichang region. The sample size was estimated using a factor of 10 relative to the number of scale items. Considering the need for exploratory and confirmatory factor analyses and an estimated 20% sample non-response rate, the projected sample sizes for the pre-survey and formal survey were 240 and 1,200 students, respectively. Samples were proportionally allocated according to each institution's enrolment size and collected through snowball sampling.

This study was approved by the Institutional Review Board (No. S202410633031) and utilized an anonymous survey. Data were used solely for academic purposes, with all participants providing informed consent and participating voluntarily. The pre-survey yielded 292 returned questionnaires, of which 217 were valid, resulting in a valid response rate of 74.32%. Following the exploratory research phase, a fully revised scale was used to conduct confirmatory factor analysis on independent samples. The formal survey yielded 1,303 returned questionnaires, of which 1,204 were valid, resulting in a valid response rate of 92.4%. Although data were collected exclusively from universities in Sichuan, students from outside the province accounted for 24.5% of the sample and represented 30 other provinces across China. This partially reflects the geographic diversity of the sample. The basic characteristics of the respondents are presented in Supplementary Table 1.

#### 3.4.3 Questionnaire quality control

Prior to questionnaire distribution, systematic training was provided to survey coordinators at participating universities to clarify questionnaire content, survey procedures, and data quality requirements. Online surveys were distributed via the Wenjuanxing platform, with real-time monitoring of response rates and data quality. Coordinators exhibiting slow progress or generating low-quality responses received targeted guidance. After data collection, invalid responses were screened using lie-detection questions and logical consistency checks. Boxplots were used to identify questionnaires with abnormal completion times, and manual review was conducted to eliminate invalid responses.

### 3.5 Data analysis

#### 3.5.1 Scale reliability and validity testing

This study used SPSS 26.0 to conduct reliability testing and exploratory factor analysis (EFA), and AMOS 24.0 to perform common method bias (CMB) testing, confirmatory factor analysis (CFA), and validity testing, thereby comprehensively evaluating the reliability and structural validity of the scale. EFA was conducted using the pre-survey sample, whereas CFA was conducted using the formal survey sample.

(1) To address potential common method bias in cross-sectional self-report data, this study employed a two-factor model approach. First, a CFA baseline model was constructed, with each item loading exclusively on its corresponding latent variable. Subsequently, a global common method factor was introduced into the baseline model, resulting in a two-factor model in which all items loaded simultaneously on both their corresponding latent variables and the common method factor. The criteria for evaluation were as follows: if, after the addition of the method factor, the increases in the Comparative Fit Index (CFI) and Tucker–Lewis Index (TLI) do not exceed 0.10, and the decreases in the Root Mean Square Error of Approximation (RMSEA) and Standardized Root Mean Square Residual (SRMR) do not exceed 0.05, this indicates is no severe common method bias (Wen et al., 2018).
(2) Reliability testing. Internal consistency reliability: Cronbach's α was used, with Cronbach's α > 0.70 for each dimension considered acceptable (Fornell and Larcker, 1981); Composite reliability (CR): a threshold of CR > 0.70 was adopted (Fornell and Larcker, 1981); Test–retest reliability: the intraclass correlation coefficient (ICC) was calculated, with ICC > 0.75 indicating good reliability (Koo and Li, 2016).
(3) Exploratory factor analysis. A KMO value > 0.70 and a significant Bartlett's test of sphericity (*p* < 0.05) were used as prerequisites for factor analysis (Kaiser, 1970). Common factor analysis with oblique rotation was employed to identify the latent factor structure. Factor loadings > 0.32, eigenvalues > 1, and communalities > 0.40 were used as criteria for item retention. Parallel analysis was also conducted, whereby factors were retained only when their observed eigenvalues exceeded those generated from a random dataset. This study is theory-driven; the identification of factors and items was guided by the theoretical framework, while the statistical results served as a reference (Worthington and Whittaker, 2006; Carpenter, 2018).
(4) Confirmatory factor analysis. CFA was conducted to assess the fit of the seven-factor theoretical structure to the formal survey data. Model fit was evaluated using the following criteria: χ^2^*/df* < 5, RMSEA < 0.08, CFI > 0.90, TLI > 0.90, and SRMR < 0.08 (Hu and Bentler, 1999; Brown, 2015; Sathyanarayana and Mohanasundaram, 2024).
(5) Validity testing. Convergent validity: assessed using Average Variance Extracted (AVE), with AVE > 0.5 considered acceptable (Fornell and Larcker, 1981); Discriminant validity: evaluated using the Fornell–Larcker criterion, requiring that the square root of the AVE for each dimension exceed its correlations with other dimensions (Fornell and Larcker, 1981; Hair et al., 2019).

#### 3.5.2 Model robustness and measurement invariance tests

To evaluate the robustness and cross-population applicability of the seven-factor measurement model, three tests were conducted across cross-sample stability, sampling error stability, and cross-population measurement equivalence. All analyses were performed using AMOS 24.0.

(1) Random split-half cross-validation. Using a simple random split-half method without replacement, the full sample (*N* = 1,204) was divided into two independent subsamples of equal size (Subsample 1: *N* = 602; Subsample 2: *N* = 602). CFA was conducted separately for each subsample, and model fit was evaluated using the same criteria applied to the full sample.
(2) Bootstrap analysis. For items with factor loadings < 0.5 in the split samples (Brown, 2015), the bootstrap method was used to perform 5,000 resampling iterations with replacement. The 95% confidence intervals for standardized factor loadings in each subsample, as well as the 95% confidence intervals for differences between subsamples, were estimated to assess whether the observed variability reflected sampling error.
(3) Multi-group measurement invariance analysis. Using gender as the grouping variable, multi-group CFA was conducted to test measurement invariance between males and females, with ΔCFI ≤ 0.01 and ΔRMSEA < 0.015 used as criteria for invariance (Cheung and Rensvold, 2002).

## 4 Results

### 4.1 Developing the initial version of the scale

Through thematic analysis, 31 initial codes were identified, and the code consistency test indicated high agreement (Kappa = 0.92), as shown in Table 2. A saturation test conducted on the reserved raw materials revealed no new information, confirming code saturation. Based on the initial coding, seven themes were extracted. Simultaneously, measurement items for the fertility intention dimension were developed through a literature review and focus group discussions. These discussions were conducted to elucidate the specific scope of “significant others” within subjective norms, including parents, relatives, neighbors, friends, and the internet (Yu and Liang, 2022; Huang, 2023; Chukwudeh and Oduaran, 2024; Zhang and Zhao, 2024; Xiao and Jiang, 2026). Measurement items for the childbearing intention dimension were also developed through a literature review and focus group discussions. Finally, drawing on the literature review and focus group discussions, the first draft of the scale was developed. Among these items, Item 30 (Time Costs of Childbearing) and Item 31 (Career Development Loss) were answered separately by male and female respondents, resulting in a total of 34 scale items, as shown in Supplementary Table 2.

**Table 2:** Initial code.

Themes	Initial code
Value prioritization	1. Fertility independence, 2. Fertility necessity, 3. Pursuit of self-actualization, 4. Focus on self-indulgence
Value of children	1. Carry on the family line, 2. Providing for parents in old age, 3. Family integrity, 4. Household labor force, 5. Fulfillment in life, 6. Emotional reliance
Reproductive perspective	1. Parental pressure, 2. Relatives' gossip, 3. Neighbors' criticism, 4. Friends' attitudes, 5. Online opinion
Parenting Perspective	1. Parental financial support, 2. Parental childcare assistance, 3. National policies, 4. Employer attitudes
Self-perceived ability	1. Financial ability and career development, 2. Sense of responsibility, 3. Psychological resilience, 4. Parenting skills
Fertility pressure	1. Employment environment, 2. Cost of living and housing prices, 3. Forced self-alteration, 4. Heavy responsibility of upbringing
Fertility cost	1. Physical cost of childbirth, 2. Financial cost of raising children, 3. Time cost of childbearing, 4. Career Development Loss

### 4.2 Scale item refinement

The Cronbach's α values for the dimensions in the preliminary survey ranged from 0.513 to 0.863, and the Kaiser–Meyer–Olkin (KMO) value was 0.824. Bartlett's test of sphericity was statistically significant (*p* < 0.01), indicating that the data were suitable for factor analysis. Common factor analysis with oblique rotation was performed. A total of 7 factors were extracted, the factor loadings and communality of most items met conventional standards. The factor loadings for items D2 and H2 were slightly below the conventional threshold of 0.32; the communality for items A1, C1, D1, D2, E1, F3, and H2 was slightly below the conventional standard of 0.40. However, the target group confirmed in cognitive interviews that the aforementioned items possess significant semantic relevance and correspond to the core theoretical connotations of their respective dimensions. Removing these items based solely on statistical thresholds would compromise the scale's content validity and the completeness of construct coverage. Therefore, based on the principle of theoretical priority, the above items are retained.

Parallel analysis was conducted to assist in determining the optimal factor structure, using the criterion that observed eigenvalues should exceed the 95th percentile of randomly generated eigenvalues. Although the results supported the retention of only three principal factors, the remaining dimensions were retained because they possessed clear theoretical foundations and unique measurement significance based on prior research and the conceptual framework of fertility concepts. Consequently, all predefined core dimensions were ultimately retained (Fabrigar et al., 1999; Hayton et al., 2004), as shown in Supplementary Tables 3–5 and Supplementary Figure 1.

Based on the results of the exploratory factor analysis and reliability analysis, the questionnaire items were revised in conjunction with focus group discussions and cognitive interviews. The revision principles were as follows:

(1) Items with factor loadings < 0.32 and those that could not be assigned to any valid dimension were deleted or revised. For example, the original item, “My expected age for childbearing,” used predefined age categories as response options. After revision, it was changed to “My intention to have children before the age of 30,” with response options ranging from “very weak” to “very strong.” This format better reflects respondents' evolving fertility-related preferences than predefined age categories (Ning et al., 2022; Qian and Huang, 2024; Qiao et al., 2024), thereby reducing potential measurement-related cognitive bias.
(2) Items within dimensions exhibiting low Cronbach's α coefficients were revised. For example, the original item, “The frequency with which I encounter negative information online about the economic pressures, physical harm, and career obstacles associated with childbearing,” was revised to “When I see netizens discussing fertility topics, they tend to focus more on the disadvantages of fertility.” This revision avoided broad generalizations about online environments, emphasized individual experiences, and improved measurement accuracy.
(3) For items that were not clearly categorized within predefined dimensions, wording was refined to improve conceptual alignment. For example, the original item, “Among my friends, some believe not having children is better,” was initially associated with both the “Value prioritization” and “Peer and internet norms” dimensions. It was revised to “When discussing fertility topics with friends, I tend to focus more on the disadvantages of having children,” thereby shifting the emphasis toward normative perceptions and more clearly distinguishing it from the “Value prioritization” dimension.
(4) Item wording was refined to improve comprehension among college students and to adopt more neutral language, thereby reducing emotional resistance. This enabled respondents to answer from a more reflective personal perspective, improving both data objectivity and discriminative power.

### 4.3 Revised scale

#### 4.3.1 Structure and items of the revised scale

The revised scale comprises 29 items across seven dimensions: value prioritization, value of children, family and neighborhood norms, peer and internet norms, self-perceived ability, pressure and cost, and fertility intention, as shown in Supplementary Table 6. The value prioritization dimension assesses the relative importance individuals assign to “self-actualization and fulfillment” vs. “fulfilling reproductive responsibilities”; the value of children dimension evaluates perceptions of the instrumental and emotional value of children; the family and neighborhood norms dimension captures expectations and pressures related to reproductive decisions from parents, older relatives, and neighbors; the peer and internet norms dimension reflects normative influences from peers, friends, relatives, and online public opinion; the self-perceived ability dimension assesses individuals' subjective evaluation of their future career prospects and economic capacity; the pressure and cost dimension measures perceived burdens associated with childbearing and child-rearing; and the fertility intention dimension evaluates the strength of fertility intentions, including overall intention, desired number of children, and preferred timing.

#### 4.3.2 Common method bias test

The baseline seven-factor model fitted the data well and met the fit criteria for the measurement model of the structural equation model (see Section 3.3.3 for details). After incorporating the global common-method factor into the baseline model, the following values were obtained: ΔCFI = 0.026, ΔTLI = 0.026, ΔRMSEA = 0.011, and ΔSRMR = 0.015. The changes in all indices complied with general guidelines for common-method bias testing (Table 3). This study does not exhibit severe common-method bias, and it will not substantially interfere with the validity and reliability testing of the scale or the core research conclusions.

**Table 3:** Common method bias test.

Comparison	CFI	TLI	RMSEA	SRMR
CFA	0.934	0.925	0.059	0.051
Bifactor model	0.960	0.951	0.048	0.036
Changes in model fit	ΔCFI	ΔTLI	ΔRMSEA	ΔSRMR
Standard	< 0.1	< 0.1	< 0.05	< 0.05
Results	0.026	0.026	0.011	0.015
Conclusion	Yes	Yes	Yes	Yes

#### 4.3.3 Confirmatory factor analysis

(1) Model Fit Test: Using AMOS 24.0 software, a confirmatory factor analysis model comprising 7 latent variables, 29 observed variables, and 29 residual variables was constructed. The standardized weights for all items were greater than 0.5, as shown in Figure 2. According to the authoritative methodological guidelines established by Hair et al. and Kline (Hair et al., 2019; Kline, 2023), RMSEA, SRMR, CFI, and TLI are the core robust fit indices currently recommended by the academic community. In this study, all of these indices achieved excellent fit levels, as shown in Table 4. GFI = 0.898, which does not meet the standard of >0.9. However, given the inherent limitations of this metric—namely, its high sensitivity to large samples and multidimensional models (Hair et al., 2019; Kline, 2023)—and considering the excellent performance of the core robust indicators, we conclude that the measurement model in this study fits well overall. At the same time, χ^2^*/df* = 5.203, which also does not meet the criterion of < 5. In large samples, chi-square values are highly sensitive to minor model discrepancies, often resulting in inflated χ^2^ scores (Sathyanarayana and Mohanasundaram, 2024). To further examine whether the model fit is affected by sample size or sampling variation, a series of robustness tests were conducted; the results are detailed in Section 4.3.5 and Supplementary Tables 7–9 and Supplementary Figures 2, 3.
(2) Validity Analysis: AVE values exceeded 0.5, demonstrating strong convergent validity for the model, as shown in Table 5. The square root of the AVE for each latent variable exceeded the correlation coefficients among other latent variables, indicating satisfactory discriminant validity. The presence of negative correlations or no correlations among the variables confirms the model's multidimensional and complex conceptual nature (Rubin et al., 2012).
(3) To determine the optimal factor structure of the scale, we compared the one-factor model, the three-factor model, and the seven-factor model using the formal survey sample. The model fit indices are presented in Table 6. The seven-factor model achieved acceptable fit indices and outperformed both the three-factor model and the one-factor model. Based on the fit indices and theoretical plausibility, we used the seven-factor model as the basis for subsequent reliability and validity tests.

**Figure 2:**
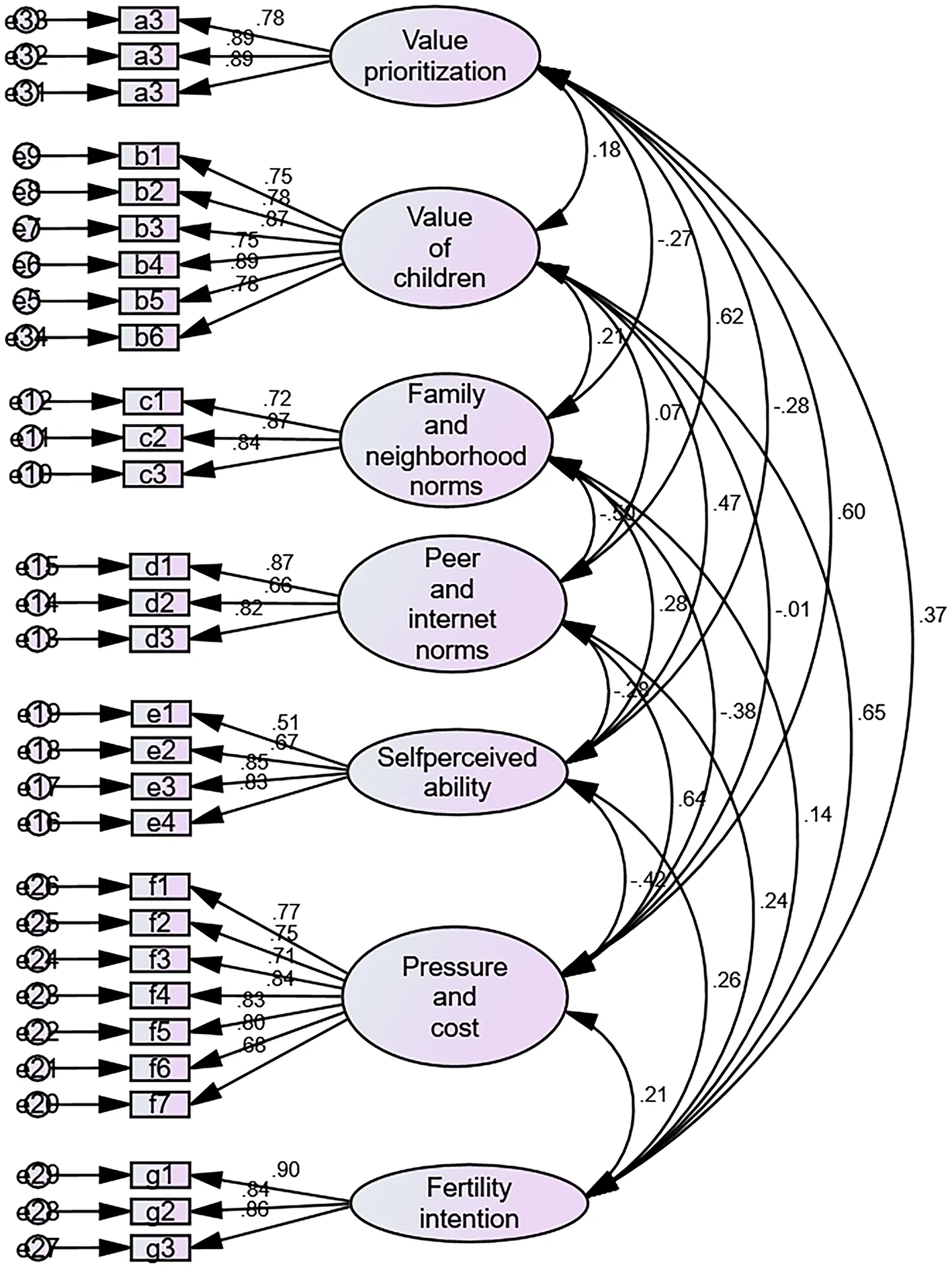
Confirmatory factor analysis model.

**Table 4:** Confirmatory factorial structure analysis.

Statistical test measure	Standard	Results	Fitting judgment
RMSEA	≤ 0.08	0.059	Yes
GFI	>0.9	0.898	No
SRMR	≤ 0.08	0.051	Yes
RFI	>0.9	0.909	Yes
TLI	>0.9	0.925	Yes
CFI	>0.9	0.934	Yes
IFI	>0.9	0.934	Yes
NFI	>0.9	0.920	Yes
χ^2^*/df*	< 5	5.203	No
PGFI	>0.5	0.735	Yes
PNFI	>0.5	0.807	Yes
PCFI	>0.5	0.819	Yes

**Table 5:** Distinguishing between validity and convergent validity.

Variable	Fertility intention	Pressure and cost	Self-perceived ability	Peer and internet norms	Family and neighborhood norms	Value of children	Value prioritization
Fertility intention	**0.867**						
Pressure and cost	0.209^**^	**0.773**					
Self-perceived ability	0.257^**^	−0.421^**^	**0.728**				
Peer and internet norms	0.241^**^	0.645^**^	−0.283^**^	**0.791**			
Family and neighborhood norms	0.137^**^	−0.383^**^	0.277^**^	−0.498^**^	**0.813**		
Value of children	0.654^**^	−0.013	0.469^**^	0.073^*^	0.207^**^	**0.805**	
Value prioritization	0.365^**^	0.601^**^	−0.282^**^	0.623^**^	−0.273^**^	0.181^**^	**0.858**
AVE	0.752	0.597	0.530	0.625	0.661	0.647	0.735

^*^p < 0.05; ^**^p < 0.01. Bold values indicate the square root of the AVE for each latent variable.

**Table 6:** Comparison of competing models.

Model	*χ^2^/df*	CFI	TLI	RMSEA	SRMR
One-factor model	38.879	0.372	0.323	0.177	0.208
Three-factor model	9.397	0.930	0.917	0.084	0.056
Seven-factor model	5.203	0.934	0.925	0.059	0.051

#### 4.3.4 Reliability analysis

The Cronbach's α coefficient for the overall scale was 0.853 (*N* = 1,204), with all dimensions exceeding 0.8, and the CR values for each dimension were all greater than 0.8. Six months after the initial survey, a convenience sample of 50 post-00s college students completed the questionnaire, followed by a retest 2 weeks later. The test–retest reliability of the Fertility Concept Scale was 0.917, with coefficients for individual dimensions ranging from 0.767 to 0.857, indicating good reliability, as shown in Table 7.

**Table 7:** Reliability analysis.

Latent variable	Number of items	Cronbacha's α (*N* = 1,204)	CR (*N* = 1,204)	ICC (*N* = 50)
Value prioritization	3	0.899	0.893	0.813
Value of children	6	0.916	0.916	0.762
Family and neighborhood norms	3	0.850	0.853	0.774
Peer and internet norms	3	0.828	0.832	0.767
Self-perceived ability	4	0.809	0.813	0.817
Pressure and cost	7	0.909	0.912	0.846
Fertility intention	3	0.901	0.901	0.857

#### 4.3.4 Model stability testing

(1) Random split-half cross-validation. The results show that the χ^2^/df values for Subsample 1 and Subsample 2 were 3.373 and 3.128, respectively. Compared with the full sample (χ^2^*/df* = 5.203), this metric decreased in both subsamples and was less than 5, meeting the commonly accepted criterion of χ^2^*/df* < 5 and confirming the inherent sample-size sensitivity of this metric. The remaining core fit indices—RMSEA, CFI, TLI, and SRMR—were similar across the two subsamples and all met the criteria for good fit (RMSEA ≤ 0.08, CFI > 0.90, TLI > 0.90, and SRMR ≤ 0.08), indicating that the seven-factor model exhibits good cross-sample stability in the randomly split subsamples of this study, as shown in Supplementary Table 7, Supplementary Figures 2, 3.
(2) Bootstrap analysis. In the two subsamples obtained by random splitting, the standardized factor loadings for Item e1 were 0.483 and 0.546, respectively. In Subsample 1, the factor loading was less than 0.5. To determine whether this variation was due to sampling error, a stability test was conducted using the bootstrap method (5,000 resamples with replacement, 95% confidence interval). The results show that the 95% bootstrap confidence interval for the factor loading of Item e1 in Subsample 1 was [0.401, 0.693], whereas the corresponding confidence interval in Subsample 2 was [0.539, 0.827], and the two confidence intervals overlapped. Simultaneously, the 95% bootstrap confidence interval for the difference in loadings of Item e1 between the two subsamples was [−0.069, 0.345], which includes 0, indicating that the difference in loadings between the two groups was not statistically significant. The observed fluctuations can therefore be attributed to sampling error, and Item e1 exhibits acceptable cross-sample stability in the randomly split subsamples of this study, as shown in Supplementary Table 8.
(3) Test of measurement invariance. This study conducted a multi-group analysis based on gender to verify the measurement invariance of the scale across gender groups. Independent confirmatory factor analyses were conducted for the two groups. The results showed that the baseline measurement models fit well for both groups (male group: χ^2^*/df* = 2.588, RMSEA = 0.063, CFI = 0.928, TLI = 0.918, SRMR = 0.054; female group: χ^2^*/df* = 3.894, RMSEA = 0.060, CFI = 0.929, TLI = 0.919, SRMR = 0.053), meeting the prerequisites for measurement invariance testing. The invariance test results indicate that the changes in fit indices between adjacent nested models met the criteria (ΔCFI ≤ 0.01, ΔRMSEA < 0.015), suggesting that the scale exhibits full measurement invariance between male and female groups and that the measurement results are suitable for cross-gender comparisons, as shown in Supplementary Table 9.

In summary, although χ^2^/df was slightly elevated in the full sample, the results of random split-half cross-validation, bootstrap analysis, and measurement invariance testing all support the robustness of the seven-factor model.

## 5 Discussion

### 5.1 Interpretation of the structure of fertility concepts

This study constructed a theoretical framework integrating TPB and SNT. Through mixed-method research, it developed a scale measuring the fertility concepts of Chinese college students born in the “post-00s” generation, revealing the multidimensional structure of this group's fertility concepts. The seven-factor structure identified in this study does not fully align with the three core constructs of the original TPB; rather, it represents a contextualized extension of the theory when applied to a specific population and domain. While TPB provided the theoretical framework for scale design, the theory exhibits a more refined structure when applied specifically to fertility concepts among post-00s college students. Unlike scales that quantify individual fertility propensity using total scores (Naghibi et al., 2019; Huczewska et al., 2023; Cheng et al., 2025), this scale, similar to the Attitudes toward Fertility and Childbearing Scale (AFCS) developed by Söderberg et al. (2013), focuses on analyzing the complexity and heterogeneity of core concepts. This approach reveals the multidimensional structure of fertility concepts and provides a foundation for analyzing the mechanisms underlying fertility intentions. This multidimensional structure not only enriches the conceptual depth of fertility perspectives but also addresses the limitations of previous research, which often suffered from single-dimensionality, conceptual gaps, or dimensional confusion (Qiao et al., 2024; Zou and Lai, 2024; Xiao et al., 2025), and compensates for the limitations of fertility attitude studies that emphasized descriptive phenomena while neglecting multidimensional deconstruction. At the same time, this scale is suitable for use across gender groups, addressing the limitations of single-gender scales used in the studies by Qiao et al. (2024) and Söderberg et al. (2013), and meeting the needs of comparative research across different gender groups. Furthermore, unlike most studies that explicitly distinguish between fertility concept and fertility intention (Liu et al., 2021; Chen, 2025), this study adopts an integrated perspective by treating fertility intention as an integral component of the multidimensional structure of fertility concepts. Intention links to other dimensions of concepts. Embedding fertility intentions within the conceptual framework addresses the fragmented understanding of fertility concept dimensions in existing research.

### 5.2 Reflection of the generational distinctiveness of post-00s college students' fertility concepts

Compared with previous studies, the scale developed in this study better reflects the generational distinctiveness of post-00s college students' fertility concepts and is more aligned with their communication patterns.

(1) The value prioritization dimension complements the value of children dimension. Value prioritization reflects post-00s college students' increased emphasis on self-worth, which is a key characteristic distinguishing this group from previous generations (Mao et al., 2024). The value of children dimension reflects the coexistence and interaction of instrumental and emotional values. This finding aligns with previous research indicating that “while the instrumental value of children has faced significant challenges, it has not disappeared,” underscoring the continued influence of traditional Chinese family culture (Yang and Wu, 2021; Ning et al., 2022). At the same time, this study employs more neutral and objective items, such as “Children can extend their parents' lives,” thereby avoiding the problems encountered in previous studies, where statements such as “Having a son is for the sake of continuing the family line” tended to trigger cognitive dissonance and emotional responses, resulting in measurement bias (Mao et al., 2024; Xu and Guo, 2024).
(2) Dividing subjective norms into family and neighborhood norms (closely linked to traditional norms) and peer and internet norms (closely linked to modern norms) addresses the limitations of previous studies that focused on a single normative source (Chukwudeh and Oduaran, 2024; Sahraei et al., 2024) and better reflects the complex social environment of post-00s university students (Lian, 2025). In particular, the inclusion of internet norms reflects the reality that post-00s individuals are “digital natives” (Li et al., 2022), providing a basis for thematic analysis using web data.
(3) Previous studies of married populations have largely focused on the role of available resources in fertility behavior (Ning et al., 2022; Qiao et al., 2024), whereas the self-perceived ability dimension in this study emphasizes students' subjective assessments of their future reproductive potential in terms of career development, economic capacity, family responsibilities, and caregiving ability. Because this group is currently in the career-planning stage and has not yet encountered actual fertility decisions, subjective expectations are more appropriate than objective measures of available resources.
(4) The pressure and cost dimension builds on previous research addressing economic and psychological pressures (Billari et al., 2009; Naghibi et al., 2019) and further incorporates key concerns of post-00s college students shaped by contemporary economic and social contexts, as well as generational tendencies toward individualism. These concerns include trade-offs among childbearing, career development, leisure activities, and lifestyle preferences (Ke et al., 2023), thereby improving population-specific measurement precision.
(5) The fertility intention dimension consists of three items assessing overall fertility intention, numerical intention, and timing intention, respectively, with response options standardized from “very weak” to “very strong.” This design not only maintains logical consistency within the dimension but also aligns the measurement of fertility intentions with that of the other dimensions. This approach, which focuses on the strength of fertility intention, better reflects respondents' evolving views on childbearing than response options based on predetermined numbers of children or childbearing ages (Ning et al., 2022; Qian and Huang, 2024; Qiao et al., 2024), thereby helping to reduce measurement-related cognitive bias.

### 5.3 Exploring a scale development approach based on web data thematic analysis

This study explored a respondent-centered scale development approach that generates initial items through web data collection and thematic analysis and subsequently optimizes these items through cognitive interviews with the target population. Compared with traditional scale-development paradigms, this approach demonstrates greater adaptability and methodological validity in measuring college students' fertility concepts. During the item-generation phase, thematic analysis based on web texts avoids limitations associated with traditional methods, such as insufficient sample representativeness and high implementation costs. Existing scales are typically developed through in-depth interviews with relatively small samples, which are often constrained by sample size and homogeneity and therefore may not fully capture the diversity and heterogeneity of fertility-related perceptions among young people. The web-text approach adopted in this study overcomes the sample-coverage limitations of traditional face-to-face interviews and enables the inclusion of native narratives from a broader and more heterogeneous population (Kozinets, 2002). Moreover, spontaneous expressions in anonymous online environments also circumvent social desirability bias commonly observed in interview settings and are consistent with the behavioral characteristics of post-00s college students as “digital natives” (Hu et al., 2022). Thematic analysis based on web data therefore enhances content coverage and cultural relevance, consistent with the principle that scale items should be grounded in the authentic experiences of the target population (Boateng et al., 2018).

During item optimization and content-validity assessment, cognitive interviews with the target population were used to directly examine respondents' comprehension processes, response logic, and interpretive biases. This approach systematically verified the consistency between item interpretation and construct definitions, thereby reducing systematic measurement errors arising from ambiguity and wording bias. It represents a core methodological strategy for ensuring measurement validity in scale development (Scott et al., 2021). Furthermore, group self-report during cognitive interviews can compensate for poor item fit resulting from intergenerational cognitive bias when expert consultation is applied to topics with pronounced generational differences, such as fertility concept.

### 5.4 Value and limitations

The value of this study lies in three dimensions: theoretical, methodological, and practical. At the theoretical level, this study clarifies the core connotations and multidimensional structure of fertility concepts among Chinese college students born in the post-00s generation. It establishes analytical dimensions aligned with the generational characteristics of this group, thereby providing a standardized, integrative analytical perspective, and a reusable theoretical framework for research on fertility concepts. At the methodological level: (1) a questionnaire was developed and preliminarily validated, providing a measurement tool to advance research on fertility concepts among post-00s college students from descriptive analysis toward investigation of underlying mechanisms; (2) a respondent-centered approach to scale development was established, combining collection of publicly available online data, thematic analysis, and cognitive interviews. This approach is suitable for developing scales for sociopsychological constructs with strong generational characteristics and contextual dependence, as well as for highly active online populations, thereby offering methodological reference for related studies. At the practical level, this study provides a foundation of measurement tools for the dynamic monitoring of fertility concept among post-00s college students.

Due to limitations in study design and implementation, several constraints remain. (1) As a preliminary validation study, although we have examined the scale's structural validity, internal consistency, and test–retest reliability, to control the number of scale items, reduce participants' response burden, lower the refusal rate, and improve overall data quality, the questionnaire did not include measures of marriage attitudes, gender-role attitudes, family fertility expectations, or a mature fertility scale. Consequently, criterion validity was not examined, resulting in insufficient evidence regarding external validity; (2) Although the sample included respondents from 31 provinces, autonomous regions, and municipalities, it was predominantly drawn from Sichuan Province, and the regional distribution was unbalanced, limiting the generalizability of the findings at the national level; (3) Because the scale was developed using online data, the demographic characteristics of all commenters could not be verified, potentially resulting in the inclusion of non-target groups and the exclusion of students who are less active on social media. In addition, platform-specific characteristics such as anonymity may encourage performative expressions that do not fully reflect individuals' actual views. Furthermore, social media algorithms may amplify emotional or extreme viewpoints, meaning that the extracted text data may not fully represent individuals' true opinions. Although cognitive interviews were conducted to calibrate item development, the limited sample size of these interviews restricted the range of perspectives that could be captured and could not completely eliminate bias in item design. Consequently, these limitations remain difficult to fully address; (4) The cross-sectional design limits the ability to capture dynamic changes in fertility concepts across policy shifts, socioeconomic transitions, and individual life-course stages, thereby constraining the assessment of temporal sensitivity.

To address these limitations, future research could focus on the following directions: (1) Incorporating measures of marriage attitudes, gender-role attitudes, family fertility expectations, and mature fertility scales into the assessment framework to evaluate external validity; (2) Expanding the sample to include more provinces and a wider range of university types, thereby enabling assessment of nationwide applicability and cross-regional measurement invariance; (3) Conducting face-to-face interviews to verify demographic characteristics and include populations that are less active online, thereby refining and validating the item pool; and (4) Implementing longitudinal designs to examine the evolution of fertility concepts over time and to assess temporal stability and predictive validity.

## 6 Conclusion

Guided by an integrated theoretical framework combining TPB and SNT, this study employed a mixed-methods approach to preliminarily develop a scale measuring fertility concept among Chinese college students born in the post-00s generation. Based on preliminary validation using a sample of college students from universities in Sichuan, the scale demonstrated good reliability and validity and effectively complements existing tools in terms of dimensional completeness, population suitability, and cultural adaptability. Future research should further examine the external validity and cross-sample stability of the scale. On this basis, the scale can provides a measurement tool foundation for the routine monitoring and dynamic tracking of fertility concepts among post-00s university students in China, establishing an integrated measurement framework and a foundation for future research on the mechanisms underlying fertility intention formation.

## Data Availability

The raw data supporting the conclusions of this article will be made available by the authors, without undue reservation.
