# Supplementary material for: Development and preliminary validation of a fertility concept scale for Chinese post-00s college students based on thematic analysis
Source: Front Psychol. 2026 Sep 16;17:1841927. doi: 10.3389/fpsyg.2026.1841927 (PMC13623929; doi:10.3389/fpsyg.2026.1841927)
Supplement: Supplementary file 1 [file Table_1.DOCX]

Supplementary Material

# 1 Supplementary Tables

## 1.1 **Supplementary** Tab**le 1 Basic Information on the Survey Participants**

| Project | Category | Quantity | Percentage（%） | Project | Category | Quantity | Percentage（%） |
| --- | --- | --- | --- | --- | --- | --- | --- |
| Age | 18–20 years old | 625 | 51.9 | Family Structure | Two-parent family | 920 | 76.4 |
|  | 21–22 years old | 539 | 44.8 |  | Single-parent family (father) | 96 | 8.0 |
|  | 23–25 years old | 40 | 3.3 |  | Single-parent family (mother) | 110 | 9.1 |
| Gender | Male | 398 | 33.1 |  | Other | 78 | 6.5 |
|  | Female | 806 | 66.9 | Relationship with Mother | Very distant | 35 | 2.9 |
| Ethnicity | Han Chinese | 1094 | 90.9 |  | Somewhat distant | 50 | 4.2 |
|  | Ethnic minority | 110 | 9.1 |  | Average | 209 | 17.4 |
| Household Registration | Urban | 475 | 39.5 |  | Fairly close | 563 | 46.8 |
|  | Rural | 729 | 60.5 |  | Very close | 347 | 28.8 |
| Place of Origin | Within Sichuan Province | 909 | 75.5 | Relationship with Father | Very distant | 57 | 4.7 |
|  | Outside Sichuan Province | 295 | 24.5 |  | Somewhat distant | 95 | 7.9 |
| Educational Background | Bachelor’s degree | 776 | 64.5 |  | Average | 328 | 27.2 |
|  | Associate degree | 428 | 35.5 |  | Fairly close | 494 | 41.0 |
| Relationship Status | Have been in a relationship before, currently single | 450 | 37.4 |  | Very close | 230 | 19.1 |
|  | Have never been in a relationship | 459 | 38.1 | Relationship Between Parents | Very strained | 73 | 6.1 |
|  | Currently in a relationship | 295 | 24.5 |  | Somewhat strained | 104 | 8.6 |
| Monthly Disposable Income | 1,500 yuan or less | 391 | 32.5 |  | Neutral | 385 | 32.0 |
|  | 1,501–2,000 yuan | 452 | 37.5 |  | Fairly affectionate | 413 | 34.3 |
|  | 2,001–2,500 yuan | 161 | 13.4 |  | Very affectionate | 229 | 19.0 |
|  | 2,501–3,000 yuan | 121 | 10.0 | Only Child | Yes | 375 | 31.1 |
|  | 3,000 yuan and above | 79 | 6.6 |  | No | 829 | 68.9 |
| Family  Housing Situation | Own home | 900 | 74.8 | Relationship with Siblings | (No answer) | 375 | 31.1 |
|  | Rent | 114 | 9.5 |  | Very poor | 14 | 1.2 |
|  | Stay in a borrowed home | 66 | 5.5 |  | Not very good | 22 | 1.8 |
|  | Other | 124 | 10.3 |  | Average | 146 | 12.1 |
| Family  Social Status | 1 point | 147 | 12.2 |  | Fairly good | 330 | 27.4 |
|  | 2 points | 300 | 24.9 |  | Very good | 317 | 26.3 |
|  | 3 points | 525 | 43.6 |  |  |  |  |
|  | 4 points | 144 | 12.0 |  |  |  |  |
|  | 5 points | 88 | 7.3 |  |  |  |  |

## 1.2 **Supplementary** Tab**le 2 Initial version scale item**

| Theme | Initial version scale item | References |
| --- | --- | --- |
| Value prioritization | 1.The decision to have children should be based on personal intention, free from external interference | (Mönkediek & Bras, 2018) |
|  | 2.Fertility is an indispensable experience in life | (Mönkediek & Bras, 2018) |
|  | 3.Achieving self-worth is more important than starting a family and raising children | (Tian et al., 2022) |
|  | 4.Personal enjoyment and freedom are more important than raising children | (Tong & Wang, 2024) |
| Value of children | 5.children can extend their parents’ lives | (Feng & Nie, 2024) |
|  | 6.Children can enhance parents' financial security in their old age | (von Saenger et al., 2023) |
|  | 7.Children can make families more complete and stable | (Luppi & Mencarini, 2018) |
|  | 8.Children can contribute to the family as labor force when they grow up | (Sheng & Li, 2023) |
|  | 9.Children can bring greater fulfillment to parents’ lives | (Nelson et al., 2014) |
|  | 10.Children can serve as emotional support for parents | (Xiao, 2022) |
| Reproductive perspective | 11.My parents consider not having children to be utterly unfilial | (Jin et al., 2018) |
|  | 12.My relatives gossip about people who don’t have children | (Wang et al., 2022) |
|  | 13.My neighbors judge people who don't have children | (Wang et al., 2022) |
|  | 14.Among my friends, some believe not having children is better | (Liang & Guan, 2024) |
|  | 15.The frequency with which I encounter negative information online about the economic pressures, physical harm, and career obstacles associated with fertility | (C. Ning et al., 2022) |
| Parenting  perspective | 16. My parents’ stance on the view that “parents should financially support their children in childbirth and child-rearing” | (Park et al., 2010) |
|  | 17.My parents' stance on the view that “parents should assist their children in childcare” | (Cheng & Hsu, 2020) |
|  | 18. I believe the actual level of support provided by the country's current policies for childbirth | (Zhang et al., 2025) |
|  | 19. I believe the actual level of support provided by employers for female employees' childbirth needs | (Li et al., 2025) |
| Self-perceived  ability | 20.Compared to my peers, I have greater potential in career development and financial capability | (Shi & Wang, 2024) |
|  | 21.Compared to my peers, I demonstrate stronger family responsibility and commitment | (Gao, 2022) |
|  | 22.Compared to my peers, I possess greater compassion, patience, and tolerance | (X. Wang et al., 2024) |
|  | 23.Compared to my peers, I am more adept at caring for others. | (Yu et al., 2023) |
| Fertility  pressure | 24.Earning money is becoming increasingly difficult | (Yu et al., 2023) |
|  | 25.Having the ideal life is getting harder and harder | (Nie et al., 2025) |
|  | 26.Having and raising children forces parents to change their lifestyle habits, hobbies, and other aspects | (Chen & Li, 2024) |
|  | 27.Raising children requires constant concern for their physical and mental health, academic performance, and other areas | (Chen et al., 2024) |
| Fertility  cost | 28.Fertility inflicts significant physical and mental harm on women | (Qiao et al., 2024) |
|  | 29.Raising children entails substantial financial costs | (Zeng & Gao, 2023) |
|  | 30-1.Child-rearing consumes nearly all of a mother’s leisure and recreational time | (Kim & Yi, 2024) |
|  | 30-2.Child-rearing consumes nearly all of a father’s leisure and recreational time | (Kim & Yi, 2024) |
|  | 31-1.Fertility and child-rearing drastically reduce women’s career advancement and promotion opportunities | (Ma, 2022) |
|  | 31-2.Fertility and child-rearing drastically reduce men’s career advancement and promotion opportunities | (Ma, 2022) |
| Fertility intention | 32.My fertility intention | Focus Group Discussio(Zheng, 2014) |
|  | 33.My expected age for childbearing |  |
|  | 34.My desired number of children |  |

## 1.3 **Supplementary** Tab**le 3 Preliminary Reliability Analysis**

| Dimension | Number of items | Cronbacha’s α |
| --- | --- | --- |
| Value prioritization | 3 | 0.648 |
| Value of children | 6 | 0.863 |
| Family and neighborhood norms | 3 | 0.792 |
| Peer and internet norms | 2 | 0.513 |
| Self-perceived ability | 4 | 0.740 |
| Pressure and cost | 7 | 0.783 |
| Fertility intention | 3 | 0.604 |

## 1.4 **Supplementary** Tab**le 4 KMO and Bartlett's Sphericity Test Results from the Preliminary Analysis**

| KMO and Bartlett's test | | |
| --- | --- | --- |
| KMO | 0.824 | |
| Bartlett's Test of Sphericity | Approximate chi-square | 2453.086 |
|  | Degrees of freedom | 378 |
|  | Significance | 0.000 |

## 1.5 **Supplementary** Tab**le 5 Preliminary factor load matrix after rotation**

| Dimension | Item | communalities | Factor 1 | Factor 2 | Factor 3 | Factor 4 | Factor 5 | Factor 6 | Factor 7 |
| --- | --- | --- | --- | --- | --- | --- | --- | --- | --- |
| Value prioritization | A1 | .188 | .339 |  |  |  |  |  |  |
|  | A2 | .524 | .614 |  |  |  |  |  |  |
|  | A3 | .702 | .914 |  |  |  |  |  |  |
| Value of children | B1 | .489 |  | .634 |  |  |  |  |  |
|  | B2 | .471 |  | .633 |  |  |  |  |  |
|  | B3 | .657 |  | .788 |  |  |  |  |  |
|  | B4 | .533 |  | .832 |  |  |  |  |  |
|  | B5 | .643 |  | .705 |  |  |  |  |  |
|  | B6 | .599 |  | .727 |  |  |  |  |  |
| Family and neighborhood norms | C1 | .363 |  |  | .573 |  |  |  |  |
|  | C2 | .716 |  |  | .840 |  |  |  |  |
|  | C3 | .701 |  |  | .850 |  |  |  |  |
| Peer and internet norms | D1 | .281 | .392 |  |  |  |  |  |  |
|  | D2 | .250 | .287 |  |  |  |  |  |  |
| Self-perceived ability | E1 | .194 |  |  |  | .490 |  |  |  |
|  | E2 | .603 |  |  |  | .704 |  |  |  |
|  | E3 | .495 |  |  |  | .611 |  |  |  |
|  | E4 | .582 |  |  |  | .741 |  |  |  |
| Pressure and cost | F1 | .515 |  |  |  |  | .776 |  |  |
|  | F2 | .676 |  |  |  |  | .876 |  |  |
|  | F3 | .349 |  |  |  |  | .400 |  |  |
|  | F4 | .595 |  |  |  |  | .762 |  |  |
|  | F5 | .538 |  |  |  |  | .525 |  |  |
|  | F6 | .507 |  |  |  |  |  | .659 |  |
|  | F7 | .585 |  |  |  |  |  | .763 |  |
| Fertility intention | H1 | .716 |  |  |  |  |  |  | .701 |
|  | H2 | .147 |  |  |  |  |  | .254 |  |
|  | H3 | .721 |  |  |  |  |  |  | .840 |

## 1.6 **Supplementary** Tab**le 6 Revised Scale**

| Dimension | Item Content |
| --- | --- |
| Value prioritization | 1. Prioritizing my own quality of life over spending money on my children is more important |
|  | 2. Achieving self-worth is more important than raising children |
|  | 3. Personal enjoyment and freedom are more important than raising children |
| Value of children | 4. Children can extend their parents’ lives |
|  | 5. Children can enhance parents' financial security in their old age |
|  | 6. Children can make families more complete and stable |
|  | 7. Children can contribute to the family as labor force when they grow up |
|  | 8. Children can bring greater fulfillment to parents’ lives |
|  | 9. Children can serve as emotional support for parents |
| Family and neighborhood norms | 10. My parents would criticize people who don't have children |
|  | 11. My older relatives would gossip about people who don't have children |
|  | 12. My neighbors judge people who don’t have children |
| Peer and internet norms | 13. When I see netizens discussing fertility topics, they tend to focus more on the disadvantages of fertility |
|  | 14. When discussing fertility topics with my peer relatives, we tend to focus more on the disadvantages of fertility |
|  | 15. When discussing fertility topics with friends, we tend to focus more on the disadvantages of fertility |
| Self-perceived ability | 16. Compared to my peers, I have greater potential in career development and financial capability |
|  | 17. Compared to my peers, I demonstrate stronger family responsibility and commitment |
|  | 18. Compared to my peers, I possess greater compassion, patience, and tolerance |
|  | 19. Compared to my peers, I am more adept at caring for others. |
| Pressure and cost | 20. Earning money is becoming increasingly difficult |
|  | 21. Having the ideal life is getting harder and harder |
|  | 22. Having and raising children forces parents to change their lifestyle habits, hobbies, and other aspects |
|  | 23. Raising children requires constant concern for their physical and mental health, academic performance, and other areas |
|  | 24. Raising children entails substantial financial costs |
|  | 25. Child-rearing consumes nearly all of a mother/father’s leisure and recreational time |
|  | 26. Fertility and child-rearing drastically reduce women/men’s career advancement and promotion opportunities |
| Fertility intention | 27. My fertility intention |
|  | 28. My intention to have two or more children |
|  | 29. My intention to have children before the age 30 |

## 1.7 **Supplementary** Tab**le 7** Fit Test for the Randomized Split-Half Model

| Statistical test measure | Standard | Results（N1=602） | Results（N2=602） | Fitting judgment |
| --- | --- | --- | --- | --- |
| χ²/df | ＜5 | 3.373 | 3.128 | Yes |
| RMSEA | ≤0.08 | 0.063 | 0.06 | Yes |
| CFI | ＞0.9 | 0.929 | 0.931 | Yes |
| TCI | ＞0.9 | 0.919 | 0.921 | Yes |
| SRMR | ≤0.08 | 0.054 | 0.057 | Yes |

## 1.8 **Supplementary** Tab**le 8** Confidence Intervals Using the Resampling Self-Extraction Method

| Sample | Sample Size | Standardized Factor Loadings | 95% Bootstrap Confidence Interval |
| --- | --- | --- | --- |
| Subsample 1 | 602 | 0.483 | [0.401, 0.693] |
| Subsample 2 | 602 | 0.546 | [0.539, 0.827] |

## 1.9 **Supplementary** Tab**le 9** Test of measurement invariance

| Model | CMIN | DF | CMIN/DF | CFI | TLI | RMSEA | ΔCFI | ΔRMSEA |
| --- | --- | --- | --- | --- | --- | --- | --- | --- |
| Unconstrained | 2307.717 | 712 | 3.24 | 0.929 | 0.919 | 0.043 | - | - |
| Measurement weights | 2365.266 | 734 | 3.22 | 0.927 | 0.919 | 0.043 | 0.002 | 0.000 |
| Structural covariances | 2473.359 | 762 | 3.25 | 0.924 | 0.919 | 0.043 | 0.003 | 0.000 |
| Measurement residuals | 2699.031 | 791 | 3.41 | 0.915 | 0.912 | 0.045 | 0.009 | 0.002 |

# 2 Supplementary Figures

|  | Actual Eigenvalue | 95th Percentile Random Eigenvalue |
| --- | --- | --- |
| 1 | 5.468 | 1.7358 |
| 2 | 4.937 | 1.6155 |
| 3 | 2.173 | 1.5366 |
| 4 | 1.4 | 1.47 |
| 5 | 1.252 | 1.4035 |
| 6 | 1.196 | 1.3466 |
| 7 | 1.043 | 1.2924 |
| 8 | 0.959 | 1.2414 |
| 9 | 0.939 | 1.1903 |
| 10 | 0.866 | 1.1463 |
| 11 | 0.823 | 1.1044 |
| 12 | 0.661 | 1.06 |
| 13 | 0.637 | 1.0202 |
| 14 | 0.61 | 0.9792 |
| 15 | 0.547 | 0.9386 |
| 16 | 0.524 | 0.904 |
| 17 | 0.477 | 0.866 |
| 18 | 0.452 | 0.8301 |
| 19 | 0.424 | 0.7943 |
| 20 | 0.382 | 0.7596 |
| 21 | 0.364 | 0.7213 |
| 22 | 0.338 | 0.6895 |
| 23 | 0.307 | 0.6556 |
| 24 | 0.281 | 0.6178 |
| 25 | 0.277 | 0.5816 |
| 26 | 0.239 | 0.5451 |
| 27 | 0.23 | 0.5021 |
| 28 | 0.195 | 0.4522 |

**Supplementary Figure 1 Parallel Analysis**

**Supplementary Figure 2 Randomized split-half confirmatory factor analysis of subsample 1**

**Supplementary Figure 3 Randomized split-half confirmatory factor analysis of subsample 2**
